# First progeria monkey model generated using base editor

**DOI:** 10.1007/s13238-020-00765-z

**Published:** 2020-07-29

**Authors:** Pradeep Reddy, Yanjiao Shao, Reyna Hernandez-Benitez, Estrella Nuñez Delicado, Juan Carlos Izpisua Belmonte

**Affiliations:** 1 Gene Expression Laboratory, Salk Institute for Biological Studies, 10010 North Torrey Pines Road, La Jolla, CA 92037 USA; 2 Universidad Católica San Antonio de Murcia (UCAM), Campus de los Jerónimos, Nº 135 12, Guadalupe, 30107 Spain

In this issue, Wang et al. report on the generation of a non-human primate model of Hutchinson-Gilford progeria syndrome (HGPS) using a base editor. Base editing is an emerging novel genome editing technique for modifying a single base pair at specific sites in the genome. Base editors (BEs) have two principal components, a catalytically inactive or single strand cleaving Cas-variant, which binds to the guide RNA and a nucleobase deaminase domain to convert specific base pairs at the target loci (Komor et al., 2016; Nishida et al., 2016; Gaudelli et al., 2017). Cytosine base editor (CBE) and adenine base editor (ABE) are two base-editors, which convert Cytosine-Guanine (C-G) to Thymine-Adenine (T-A) and A-T to G-C, respectively. Likewise, RNA base editor (RBE) was created by fusing nucleobase deaminases with the Cas13 protein, which allows for a base substitution of A to inosine (I) or C to uracil (U) in the targeted RNA (Cox et al., 2017). Recently, a dual base editor was developed that can catalyze both cytosine and adenine base conversions at the same time, broadening base editing capability (Zhang et al., 2020).

Compared to other gene-editing approaches, BEs have several advantages 1) they can induce a single base-pair substitution precisely and efficiently at the targeted loci, making BE an ideal candidate for correcting pathogenic mutations, 2) they have a higher editing efficiency without the necessity of a DNA template and 3) single nucleotide conversions do not require any double-stranded breaks (DSBs), which may lead to frequent insertions and deletions or larger-scale genomic rearrangements (Ceccaldi et al., 2016; Liang et al., 2019). For these reasons, CRISPR-guided base editing is particularly attractive and has been widely applied to generate point mutations in animal models and to correct genetic mutations.

For the first time, Wang et al., have generated a non-human model of HGPS in the monkey using the newly developed base editor. HGPS is a rare disorder caused by a mutation in *LMNA*, which leads to a premature cryptic splicing site and production of a truncated protein called progerin (Eriksson et al., 2003). The truncated protein lacks the endoproteolytic cleavage site and prevents the removal of a farnesylated tail. Eventually, the accumulation of abnormal protein on the nuclear envelope leads to the manifestation of phenotypes such as nuclear abnormalities, increased DNA damage, and loss of epigenetic marks. Although the use of farnesyltransferase inhibitors to slow down this process showed some promising results in animal models, the long-term effects from the use of inhibitors are still under study (Fong 2006; Young et al., 2013).

During the last several years, the use of HGPS patient-derived fibroblasts and induced pluripotent stem cells (iPSCs) have greatly advanced our understanding of cellular and molecular defects caused by the accumulation of progerin (Zhang et al., 2011; Liu et al., 2011). Interestingly, due to the absence of Lamin A/C and progerin expression in pluripotent stem cells, the nuclear abnormalities and epigenetic alterations were not observed. However, differentiation of iPSCs to smooth muscle cells (SMCs), the primary cell type affected in the patients, lead to the reappearance of cellular defects due to the re-expression of the *LMNA* gene (Liu et al., 2011). Similarly, isogenic human stem cell lines for HGPS and Werner syndrome were created using a genome editing approach to compare the aging kinetics (Wu et al., 2018).

To better understand the pathological phenotypes of LMNA splicing, a genetically modified mouse model carrying the *Lmna A* mutation similar to humans was generated (Osorio et al., 2011). These mice showed several phenotypes observed in human HGPS, including shortened lifespan and cardiovascular abnormalities. Moreover, these mice phenocopied the HGPS phenotypes better than other mouse models, such as *Zmpste24*-deficient mice. Although the mice carry a *Lmna A* mutation similar to human HGPS, physiological differences between mice and humans can limit a complete understanding of mechanisms underlying this disease in humans. For this reason, a non-human primate model genetically engineered to develop HGPS was much needed for basic and biomedical research.

CRISPR-Cas9 is currently the most widely used genome editing tool for performing knockout or knock-in of genes. It was also successfully used in monkey zygotes to generate genetically modified animals (Niu et al., 2014; Wan et al., 2014; Zuo et al., 2017; Zhang et al., 2018). Nevertheless, a low rate of homologous recombination of the donor DNA template limits the introduction of specific genetic changes, especially at the single-nucleotide level. Wang et al., demonstrate the use of base editor in zygotes of monkey and opened new avenues for the application of base editors in medicine. The authors introduced the C>T conversion in the *LMNA* gene at 1824 in *Macaca fascicularis* (cynomolgus monkey) using the recently developed base editor, BE4max, to generate an HGPS monkey model. The authors co-injected a single guide RNA and BE4max into the zygotes, which were later transferred to surrogate females. Five live births were obtained, and two monkeys died after 5-months. The sequencing results showed that all the monkeys had the expected C>T conversion at the target locus, and three monkeys were homozygous for the mutation.

Importantly, the expression of progerin was observed in different tissues from homozygous and heterozygous monkeys, and similar to human HGPS, it was highly expressed in skin, heart, and blood vessels. Moreover, HGPS monkeys were healthy at birth but failed to gain weight during their early life. Also, HGPS monkeys suffered from loss of hair and subcutaneous fat. Interestingly, similar to human HGPS patients, the mutant monkeys developed physical changes, including a prominent forehead, protuberant eyes, and hypoplastic mandible. Furthermore, mutant monkeys also had increased vascular wall fibrosis. Notably, the fibroblasts of mutant monkeys also showed a range of well-defined cellular defects such as lower proliferation, increased senescence, and loss of heterochromatin. Finally, transcriptomic analysis of skin samples from 5-month old HGPS monkeys showed upregulation of genes associated with an inflammatory response and cytokine receptors compared to the WT monkeys. Collectively, all the features observed in mutant monkeys recapitulate the clinical descriptions seen in human HGPS patients. Another clinical feature of HGPS patients is a shorter lifespan; comprehensibly, the authors need more time to report on this in the HGPS monkeys.

The HGPS monkey model developed by Wang et al. constitutes a breakthrough to understand the HGPS disease phenotypes in a higher animal that are not fully manifested in the mouse models. Moreover, different types of drugs have been tested in HGPS mouse models to restore the expression of genes and reduce progerin levels; although they have shown promising results in mice, they have not been effective in clinical trials. HGPS is a rare disease, and a small patient number is a challenge to test all the promising strategies; the HGPS monkey model developed by Wang et al. will be of paramount importance to overcome these problems.

Among the different hallmarks of aging observed in HGPS, cellular senescence is a major one, which is also seen during normal aging and is considered to contribute to the aging of the whole organism significantly (Ribes et al., 2019). To eliminate the senescent cells, several small molecules called senolytics have been identified by large-scale screening and after showing promising results in mice, they have entered clinical trials for treating age-associated diseases such as osteoarthritis (Gorgoulis et al., 2019; Ribes et al., 2019). Moreover, antisense oligonucleotides were recently tested to knockdown the telomeric non-coding RNAs, which are induced by progerin, to control the DNA damage response and cellular senescence (Aguado et al., 2019). Similarly, an antisense oligonucleotides approach was also used to reduce the levels of progerin, which led to an extension of the lifespan of HGPS mice (Osorio et al., 2011). These strategies are promising interventions to prevent senescent phenotypes and alleviate some of the HGPS symptoms.

Additionally, epigenetic dysregulation is a major hallmark of normal and premature aging that leads to the manifestation of other age-associated cellular phenotypes. Notably, cellular reprogramming of aged or HGPS fibroblasts to pluripotency reset the epigenome of the cells and erased aging hallmarks (Zhang et al., 2011; Liu et al., 2011). Similarly, short-term expression of reprogramming factors without losing cellular identity led to the restoration of histone marks and significantly increased the lifespan of HGPS mice (Ocampo et al., 2016). These results demonstrate that controlled *in vivo* partial cellular reprogramming can have a considerable beneficial effect in ameliorating HGPS phenotypes. It is tempting to speculate that by delivering the reprogramming factors using adeno-associated viruses, partial reprogramming could be performed in HGPS monkeys and may have beneficial effects similar to HGPS mice.

Correction of a disease-causing mutation *in vivo* is another promising avenue. Genome editing strategies based on the CRISPR-Cas9 system were used recently in the HGPS mouse model to correct the mutation or prevent the expression of progerin (Beyret et al., 2019; Santiago-Fernández et al., 2019; Suzuki et al., 2019). Although the frequency of *in vivo* genome editing is low, encouraging observations, including improved health and lifespan, were made in the HGPS mice. The HGPS monkeys can be an ideal model to validate and further develop *in vivo* genome editing approaches.

All previous interventions in HGPS mouse models have shown favorable results. However, for successfully translating these strategies to the clinic, it is essential to validate these data in large animal models. Along this line, HGPS monkeys are an ideal model due to similar disease features and the fact that they are physiologically closer to humans. Moreover, HGPS models are routinely used for understanding the molecular mechanisms that are responsible for the manifestation of aging phenotypes. Hence, HGPS mouse models are widely used in aging studies, but their use is routinely criticized as they do not completely recapitulate the features observed during aging. Therefore, it will be interesting to see whether HGPS monkeys develop aging phenotypes that are similar to normal aging.

In conclusion, Wang et al. have demonstrated, for the first time, the feasibility of using the base editor in monkey zygotes to introduce single-base pair changes at the targeted loci precisely and at a higher frequency. As the mouse models do not entirely recapitulate the human disease phenotypes, there is a pressing need for better models. BEs have expanded the tools for generating non-human primate models, which are invaluable for both basic and translational research.
